# The endochondral bone protein CHM1 sustains an undifferentiated, invasive phenotype, promoting lung metastasis in Ewing sarcoma

**DOI:** 10.1002/1878-0261.12057

**Published:** 2017-08-21

**Authors:** Kristina von Heyking, Julia Calzada‐Wack, Stefanie Göllner, Frauke Neff, Oxana Schmidt, Tim Hensel, David Schirmer, Annette Fasan, Irene Esposito, Carsten Müller‐Tidow, Poul H. Sorensen, Stefan Burdach, Günther H. S. Richter

**Affiliations:** ^1^ Laboratory for Functional Genomics and Transplantation Biology Children's Cancer Research Center and Department of Pediatrics Klinikum rechts der Isar Technische Universität München Comprehensive Cancer Center Munich (CCCM) German Translational Cancer Research Consortium (DKTK) Munich Germany; ^2^ Institute of Pathology Helmholtz Zentrum München ‐ German Research Centre for Environmental Health (GmbH) Neuherberg Germany; ^3^ Department of Medicine IV, Hematology and Oncology University Hospital Halle Germany; ^4^ Institute of Pathology University Düsseldorf Germany; ^5^ Department of Medicine V, Hematology, Oncology and Rheumatology University of Heidelberg Germany; ^6^ Department of Molecular Oncology British Columbia Cancer Research Centre Vancouver BC Canada

**Keywords:** CHM1, endochondral bone, Ewing sarcoma, invasion, metastasis

## Abstract

Ewing sarcomas (ES) are highly malignant, osteolytic bone or soft tissue tumors, which are characterized by EWS–ETS translocations and early metastasis to lung and bone. In this study, we investigated the role of the BRICHOS chaperone domain‐containing endochondral bone protein chondromodulin I (CHM1) in ES pathogenesis. CHM1 is significantly overexpressed in ES, and chromosome immunoprecipitation (ChIP) data demonstrate CHM1 to be directly bound by an EWS–ETS translocation, EWS‐FLI1. Using RNA interference, we observed that CHM1 promoted chondrogenic differentiation capacity of ES cells but decreased the expression of osteolytic genes such as *HIF1A*,*IL6*,*JAG1*, and *VEGF*. This was in line with the induction of the number of tartrate‐resistant acid phosphatase (TRAP ^+^)‐stained osteoclasts in an orthotopic model of local tumor growth after CHM1 knockdown, indicating that CHM1‐mediated inhibition of osteomimicry might play a role in homing, colonization, and invasion into bone tissues. We further demonstrate that CHM1 enhanced the invasive potential of ES cells *in vitro*. This invasiveness was in part mediated via CHM1‐regulated matrix metallopeptidase 9 expression and correlated with the observation that, in an xenograft mouse model, CHM1 was essential for the establishment of lung metastases. This finding is in line with the observed increase in CHM1 expression in patient specimens with ES lung metastases. Our results suggest that CHM1 seems to have pleiotropic functions in ES, which need to be further investigated, but appears to be essential for the invasive and metastatic capacities of ES.

**Abbreviations**
ABCG2: ATP‐binding cassette, subfamily G (WHITE), member 2
CHM1: leukocyte cell‐derived chemotaxin 1 (chondromodulin 1, CNMD)
DKK2: dickkopf WNT signaling pathway inhibitor 2
ES: Ewing sarcoma
HIF1A: hypoxia‐inducible factor 1, alpha subunit
IL6: interleukin 6
JAG1: jagged 1
MMP: matrix metallopeptidase
NANOG: nanog homeobox
OPN: secreted phosphoprotein 1 (SPP1)
PROM1: prominin 1
RANKL: tumor necrosis factor superfamily member 11 (TNFSF11)
TGFB1: transforming growth factor beta
TRAP: tartrate‐resistant acid phosphatase
TSS: transcription start site
VEGF: vascular endothelial growth factor receptor 1

## 1 Introduction

Ewing sarcomas (ES) are the second most common malignancy of bone and soft tissues in children and adolescents, which accounts for 10–15% of all primary bone tumors (Burchill, 2003). Genetically, ES are defined by EWS–ETS translocations encoding aberrant transcription factors presumed to induce the highly malignant phenotype of this disease (Delattre *et al*., 1994; Lessnick and Ladanyi, 2012; Mackintosh *et al*., 2010; Sorensen *et al*., 1994). Other contributing somatic mutations involved in disease development have only been observed at low frequency (Agelopoulos *et al*., 2015; Brohl *et al*., 2014; Crompton *et al*., 2014; Tirode *et al*., 2014). ES are characterized by early metastasis into lung and bone tissues. Metastasis is commonly hematogenous and related to stemness (Burdach *et al*., 2009; Richter *et al*., 2009; Schmidt *et al*., 1985). Even though prognosis for patients with ES has markedly improved during the development of multimodal therapeutic approaches, the survival rate of patients with advanced, multifocal disease is still associated with fatal outcome (Burdach *et al*., 1993, 2010; Thiel *et al*., 2011); especially, multifocal bone or bone marrow disease and the development of metastases in bones are catastrophic events in the clinical course of patients with ES (Burdach and Jurgens, 2002; Coleman, 2006; Thiel *et al*., 2016).

Based on our previous microarray analysis, we identified the dickkopf WNT signaling pathway inhibitor 2 (DKK2) critical for terminal bone development (Li *et al*., 2005) and two BRICHOS domain‐containing genes important for chondrogenic differentiation (Deleersnijder *et al*., 1996; Klinger *et al*., 2011), to be overexpressed in ES (Hauer *et al*., 2013; Staege *et al*., 2004). We demonstrated DKK2 to be an agonist of the canonical WNT/β‐catenin pathway and to be a key player in ES metastasis, bone invasiveness, and osteolysis (Hauer *et al*., 2013).

Here, we analyzed one of the BRICHOS domain‐containing genes, *leukocyte cell‐derived chemotaxin 1* (also known as *chondromodulin 1*;* CHM1*;* CNMD*), for its function in chondro‐osseous tumor growth and invasiveness. Sanchez‐Pulido *et al*. (2002) observed that the BRICHOS domain itself seems to be involved in post‐translational processing of the corresponding pro‐proteins and/or to have a chaperone‐like activity. CHM1 expression has been previously associated with chondrosarcoma and BRICHOS domain mutations in the surfactant protein C precursor have been linked to endoplasmic reticulum stress, proteasome dysfunction, and caspase 3 activation, suggesting a role for the BRICHOS chaperone domain in microenvironmental regulation (Hedlund *et al*., 2009; Sanchez‐Pulido *et al*., 2002). Under normal conditions, CHM1 is almost exclusively expressed in the cartilage and has a strong antiangiogenic function (Hiraki and Shukunami, 2000; Hiraki *et al*., 1997; Yoshioka *et al*., 2006). The secreted, mature form of the glycoprotein is a key factor in chondrocyte proliferation and development and simultaneously inhibits terminal chondrocyte hypertrophy and endochondral ossification (Klinger *et al*., 2011; Shukunami and Hiraki, 2001). These characteristics indicated that CHM1 might be important in ES malignancy, as ES progenitor cells seem to be of premature chondrogenic origin arrested at early osteo‐chondrogenic differentiation (Hauer *et al*., 2013; von Heyking *et al*., 2016; Tanaka *et al*., 2014).

In the present study, we observed that CHM1 reduced the endothelial but enhanced the chondrocytic differentiation ability of ES. CHM1 simultaneously increased the expression of several stem cell genes such as *PROM1*. Furthermore, CHM1 overexpression promoted *in vitro* invasiveness, as well as lung metastasis of ES cells in a xenograft mouse model. In line with these findings, expression of CHM1 is significantly higher in lung metastases samples of patients with ES than in samples derived from different bone localizations. This indicates CHM1 to be important for ES malignancy, especially for maintaining an undifferentiated, metastatic phenotype in ES.

## 2 Materials and methods

### 2.1 Cell lines

ES lines (MHH‐ES1, RD‐ES, SK‐ES1, SK‐N‐MC, and TC‐71), neuroblastoma lines (CHP126, MHH‐NB11, SHSY5Y, and SIMA), and pediatric human B‐cell precursor leukemic lines (cALL2, NALM6, and 697) were obtained from the German Collection of Microorganisms and Cell Cultures (DSMZ, Braunschweig, Germany). ES line VH64 was kindly provided by Marc Hotfilder (Münster University, Münster, Germany); osteosarcoma lines (HOS, HOS‐58, MG‐63, MNNG, SaOS, SJSA01, U2OS, and ZK‐58) by Jan Smida and Michaela Nathrath, Institute of Pathology and Radiation Biology (HMGU, Neuherberg, Germany). A673 was purchased from ATCC (LGC Standards, Teddington, UK). SB‐KMS‐KS1 and SB‐KMS‐MJ1 are ES cell lines that were established in our laboratory (Grunewald *et al*., 2012; Richter *et al*., 2009). Retrovirus packaging cell line PT67 was obtained from Takara Bio Europe/Clontech (Saint‐Germain‐en‐Laye, France). Cells were maintained in a humidified incubator at 37 °C in 5–8% CO_2_ atmosphere in RPMI 1640 or DMEM (both Life Technologies, Carlsbad, CA, USA) containing 10% heat‐inactivated fetal bovine serum (Biochrom, Berlin, Germany) and 100 μg·mL^−1^ gentamicin (Life Technologies). Cell lines were checked routinely for purity (e.g., EWS‐FLI1 translocation product, surface antigen or HLA phenotype) and mycoplasma contamination.

### 2.2 RNA interference (RNAi)

For transient RNA interference, cells were transfected with small interfering RNA (siRNA) as described previously (Richter *et al*., 2009). To test transfection efficiency and gene silencing, RNA was extracted and gene expression assessed by quantitative real‐time PCR. All siRNA sequences are provided in the supplementary data.

### 2.3 Constructs and retroviral gene transfer

For stable silencing of CHM1 expression, oligonucleotides were designed corresponding to the most efficient siRNA used for transient RNA interference and retroviral gene transfer was performed as described previously (Richter *et al*., 2009). The used oligonucleotides are provided in the Supporting Information (Doc. S1).

### 2.4 Quantitative Real‐time PCR (qRT‐PCR)

Total RNA was isolated and reverse‐transcribed using the High Capacity cDNA Reverse Transcription Kit (Life Technologies) according to the manufacturer's instructions. Differential gene expression was then analyzed by qRT‐PCR using TaqMan Universal PCR Master Mix and fluorescence detection with an AB 7300 Real‐Time PCR System (both Life Technologies) as described previously (Richter *et al*., 2009, 2013). Gene expression was normalized to glyceraldehyde‐3‐phosphate dehydrogenase (GAPDH). A list of used assays is provided in the Supporting Information. NTC: nontemplate control.

### 2.5 ChIP and quantitative real‐time PCR

ChIP was performed using ChIP‐IT^®^ Express Kit (Active Motif, Carlsbad, CA, USA) according to the manufacturer's instructions. In brief, 2 × 10^7^ SK‐N‐MC and TC‐71 cells, respectively, were fixed with methanol‐free formaldehyde (Life Technologies, Darmstadt, Germany) at a final concentration of 1% for 10 min. After neutralization with glycine, cells were lysed in RIPA buffer with protease inhibitors. Samples were sonicated to an average DNA length of 200–400 bp using a M220 Focused‐ultrasonicator™ (Covaris, Woburn, MA, USA). ChIP was carried out using 5 μg of anti‐FLI1 antibody (C‐19, sc‐356X; Santa Cruz) or anti‐rabbit IgG (sc‐2027X; Santa Cruz), respectively. DNA was cleaned up using IPure kit (Diagenode, Seraing, Belgium). Quantitative real‐time PCR (qPCR) using SYBR Green (Bio‐Rad, München, Germany) was performed for different loci of the CHM1 promoter and one positive control loci at −1081 bp upstream of the transcription start site (TSS) of the EZH2 promoter. FLI1 binding was normalized to IgG control antibody using the ΔΔCT method (Livak and Schmittgen, 2001).

### 2.6 Proliferation assay

Cell proliferation was determined with an impedance‐based instrument system (xCELLigence, Roche/ACEA Biosciences, Basel, Switzerland) enabling label‐free real‐time cell analysis. Briefly, 1–3 × 10^4^ cells were seeded into 96‐well plate with 200 μL media containing 10% FBS and allowed to grow up to 60 h. Cellular impedance was measured periodically every four hours and gene knockdown was monitored by qRT‐PCR.

### 2.7 Colony forming assay

Cells were seeded in duplicate into a 35‐mm plate at a density of 5 × 10^3^ cells per 1.5 mL methylcellulose‐based media (R&D Systems, Minneapolis, MN, USA) according to the manufacturer's instructions and cultured for 10–14 days at 37 °C/5% CO_2_ in a humidified atmosphere.

### 2.8 *In vitro* invasion assay

To study cell invasion, the BioCoat™ Angiogenesis System: Endothelial Cell invasion was used (BD Biosciences, San Jose, CA, USA) according to the manufacturer's instructions as described previously (Grunewald *et al*., 2012).

### 2.9 Differentiation assay

Cellular tube formation was tested by the use of a commercial Matrigel matrix assay (Biocoat; BD Biosciences) according to the manufacturer's instruction. Briefly, cells were seeded at 5 × 10^4^ cells per well in a 96‐well plate and grown at 37 °C (5% CO_2_) in a humidified atmosphere. After 16–18 h, cells were stained with 1 μg·mL^−1^ Calcein AM Fluorescent Dye (BD Biosciences) for 30 min in the dark. Cells were imaged by fluorescence microscopy by using a Nikon Eclipse TS 100 with an attached Nikon Coolpix 5400 camera (Nikon, Tokyo, Japan).

### 2.10 Elisa

An ELISA with 48 strip wells from MyBioSource (San Diego, CA, USA) to detect CHM1 levels (MBS937594) was performed according to the manufacturer's instructions.

### 2.11 Microarray analysis

Patient material was obtained from clinical studies of the Cooperative Ewing Sarcoma Study Group in Europe. All patients provided informed consent. Biotinylated target cRNA was prepared as previously described (Richter *et al*., 2009). A detailed protocol is available at www.affymetrix.com. Samples were hybridized to Affymetrix Human Gene 1.0 ST microarrays and analyzed by affymetrix software expression console (Affymetrix, High Wycombe, UK), version 1.1. For the data analysis, robust multichip average normalization was performed, including background correlation, quantile normalization, and median polish summary method. Array data were submitted at GEO (GSE45544).

### 2.12 Animal model

Immunodeficient Rag2^−/−^γc^−/−^ mice on a BALB/c background were obtained from the Central Institute for Experimental Animals (Kawasaki, Japan) and maintained in our animal facility under pathogen‐free conditions in accordance with the institutional guidelines and approval by local authorities (Regierung von Oberbayern). Experiments were performed in 6‐ to 20‐week‐old mice.

### 2.13 *In vivo* experiments

To examine *in vivo* tumorigenicity, 2 × 10^6^ ES cells and derivatives were injected subcutaneously into the inguinal region of immunodeficient Rag2^−/−^γc^−/−^ mice, and when the tumor reached 1 cm^3^, mice were sacrificed and tumor samples were analyzed.

For the analysis of *in vivo* metastatic potential, 1.5–2 × 10^6^ ES cells and derivatives were injected in a volume of 0.2 mL into the tail vein of immunodeficient Rag2^−/−^γc^−/−^ mice as described previously (Grunewald *et al*., 2012; Richter *et al*., 2009). Mice were sacrificed after five weeks, and metastatic spread was examined in individual organs.

To investigate bone invasiveness and osteolysis, mice were anesthetized with 500 mg·mL^−1^ novaminsulfon (Ratiopharm, Ulm, Germany) and isoflurane (Abbott, Abbott Park, IL, USA) and A673 or TC‐71 derivatives were injected as described previously (Hauer *et al*., 2013). Briefly, a 30‐gauge needle was introduced through the proximal tibia plateau and 2 × 10^5^ ES cells in a volume of 20 μL were injected into the medullary cavity. In all experiments, tumors and affected tissues were recovered and processed for histological analyses. Intratibial tumor formation was monitored by X‐ray radiography.

### 2.14 Histology

Murine organs were fixed in phosphate‐buffered 4% formaldehyde and embedded in paraffin; 3‐ to 5‐μm‐thick sections were stained with hematoxylin and eosin (H&E). Hind limb bones were decalcified and paraffin‐embedded; the histological analysis with H&E was complemented by quantification of tartrate‐resistant acid phosphatase (TRAP^+^)‐stained osteoclasts. All sections were reviewed and interpreted by two pathologists (J. C‐W.; F. N. or I.E.).

### 2.15 Statistical analyses

Data are mean ± SEM as indicated. Differences were analyzed by unpaired two‐tailed Student's *t*‐test as indicated using Excel (Microsoft, Redmond, WA, USA) or Prism 5 (GraphPad Software, San Diego, CA, USA); *P* values < 0.05 were considered statistically significant (*P < 0.05; **P < 0.005; ***P < 0.0005).

## 3 Results

### 3.1 CHM1 is highly expressed in Ewing sarcomas

**Figure 1:**
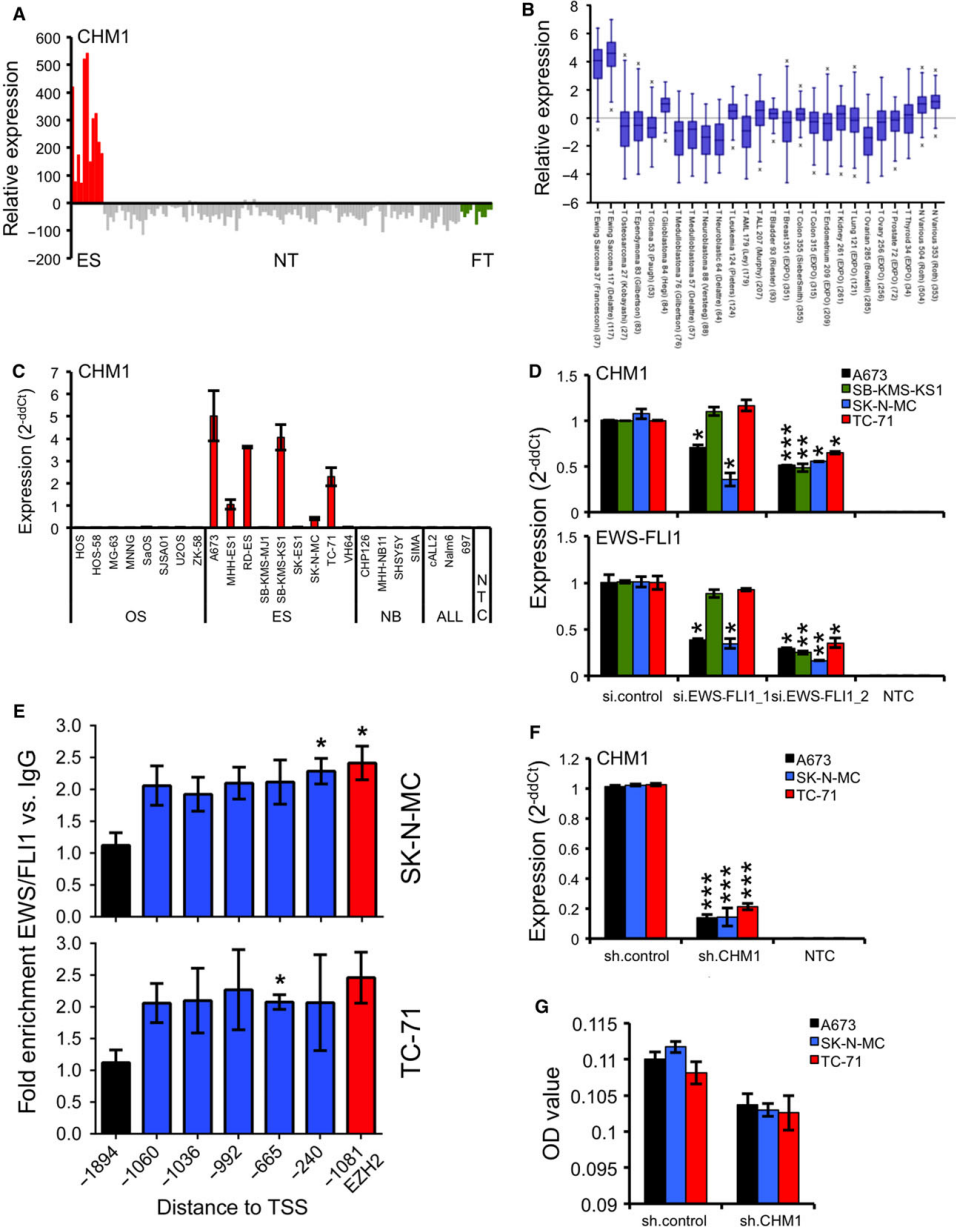
CHM1 is highly overexpressed in ES. (A) Expression profile of CHM1 in primary ES in comparison with normal tissue (NT) and fetal tissue (FT). ES, NT, and FT samples were analyzed using EOS‐Hu01 microarrays (Staege *et al*., 2004). (B) Expression levels of CHM1 in different pediatric small, round, blue cell tumors, carcinomas, and normal tissues by box plot presentation using a comparative study of the amc onco‐genomics software tool (www.amc.com). Results are 2‐log‐centered for better representation of results. The number of samples in each cohort is given in brackets. (C) CHM1 expression in different tumor cell lines analyzed by qRT‐PCR. Data are mean ± SEM. (D) RNA interference of EWS‐FLI1 expression (bottom) does reduce CHM1 expression (top). si.EWS‐FLI1_1 (less efficient) and si.EWS‐FLI1_2 represent the specific siRNAs (si.control: nonsilencing siRNA). Results of qRT‐PCR 48 h after transfection are shown. Data are mean ± SEM of two independent experiments; *t*‐test. (E) EWS‐FLI1 enrichment at the CHM1 promoter in SK‐N‐MC and TC‐71 cells. ChIP analysis was performed with FLI1 and control IgG antibodies, respectively, and analyzed by quantitative PCR for binding to different regions of the CHM1 promoter. FLI1 enrichment was detected at different ETS recognition sites −1060, −1036, −992, −665, and −240 bp upstream of the TSS of CHM1. The −1894‐bp region, which is devoid of ETS recognition sequences, served as negative control. The ETS consensus site at −1081 bp of the EZH2 promoter (Richter *et al*., 2009) was used as positive control for FLI1 binding. Data represent the mean of two independent experiments, and error bars represent standard deviations. (F) Constitutive suppression of CHM1 expression after infection of ES cells with CHM1‐specific shRNA constructs as measured by qRT‐PCR (sh.CHM1 and sh.control). qRT‐PCR data are mean ± SEM of 10 independent experiments; *t*‐test. (G) ELISA detection of CHM1 levels in the supernatant of ES cells stably transfected with CHM1 shRNA or control. Data are mean ± SEM;* t*‐test. *P < 0.05; **P < 0.005; ***P < 0.0005 (see 2.15. Statistical analyses).

Previously, we identified CHM1 to be highly expressed in ES (Staege *et al*., 2004). As shown in Fig. 1A,B, we observed high levels of CHM1 expression exclusively in ES, compared to different normal and fetal tissues (Fig. 1A), or various other pediatric or adult cancer types such as neuroblastoma, medulloblastoma, leukemia, and various carcinomas (Fig. 1B). To further validate overexpression of CHM1 in ES, we tested nine common ES cell lines against a series of different osteosarcoma, neuroblastoma, and ALL cell lines using qRT‐PCR. As expected, *CHM1* was strongly up‐regulated in ES cell lines, but not in neuroblastoma and ALL cell lines (Fig. 1C). Furthermore, analysis of mRNA levels revealed no expression of *CHM1* in osteosarcoma cell lines (Fig. 1C), while CHM1 was previously associated with inhibition of endochondral ossification (Deleersnijder *et al*., 1996; Klinger *et al*., 2011).

Subsequently, we analyzed whether the oncogenic fusion protein EWS‐FLI1 can influence CHM1 expression in four different ES cell lines. As shown in Fig. 1D, RNA interference‐mediated EWS‐FLI1 silencing led to a significant, efficiency‐dependent suppression of CHM1 levels, which indicates CHM1 expression to be associated with EWS‐FLI1. We next performed ChIP analysis with FLI1 and IgG antibodies to analyze binding of FLI1 to the CHM1 promoter. FLI1 enrichment was detected at different ETS recognition sites −1060, −1036, −992, −665, and −240 bp upstream of the TSS of CHM1 (Fig. 1E). These data suggest CHM1 to be directly regulated by the ES chimeric transcription factor, EWS‐FLI1. For subsequent analysis, we constitutively down‐regulated CHM1 in different ES cell lines (A673, SK‐N‐MC, and TC‐71) to further elucidate the influence of this gene on ES pathogenesis (Fig. 1F,G).

### 3.2 CHM1 influences the endothelial as well as chondrocytic differentiation potential of ES

**Figure 2:**
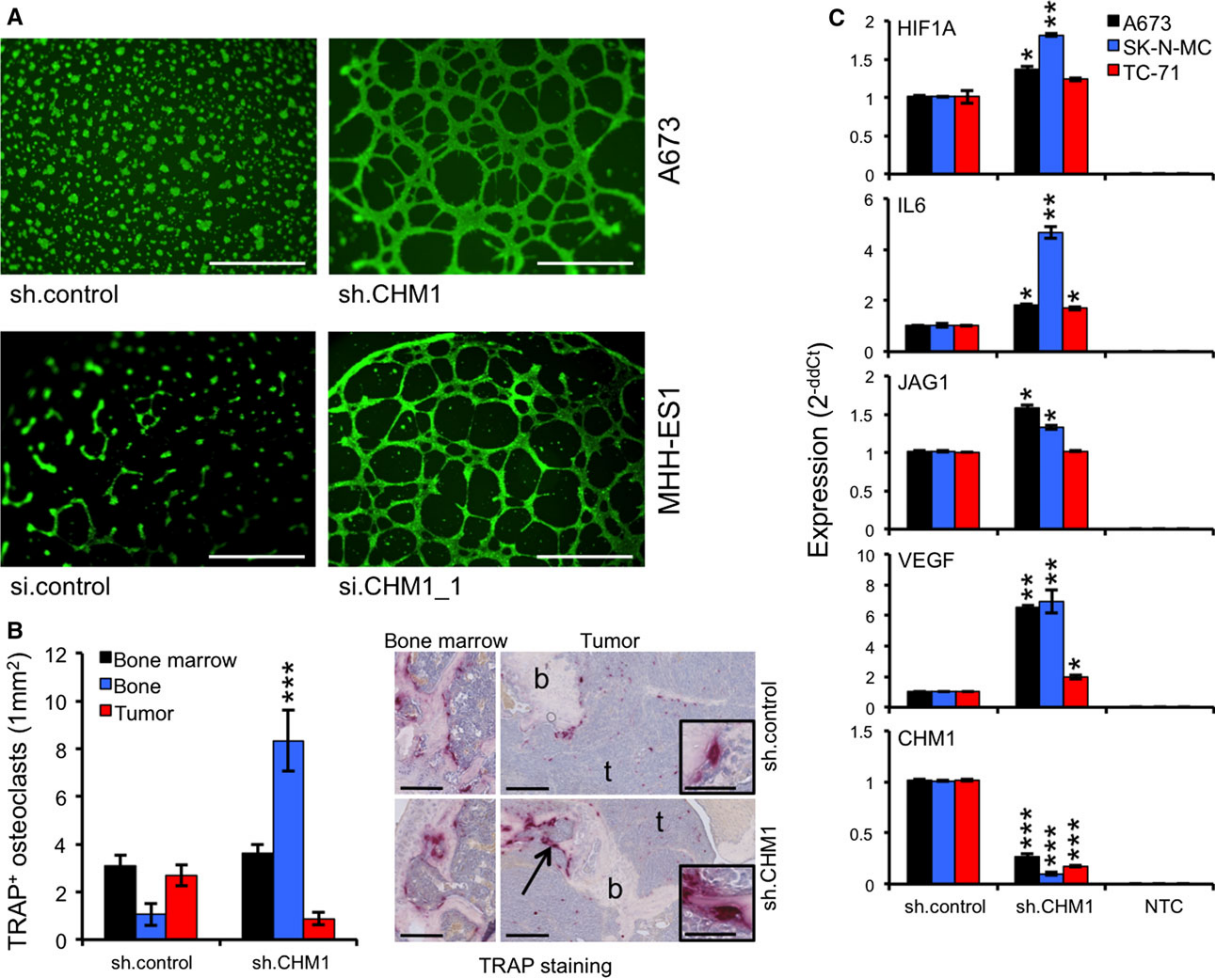
CHM1 inhibits tube formation and influences osteomimicry. (A) Tube formation assay with constitutively transfected A673 (sh.control and sh.CHM1) and transiently transfected MHH‐ES1 (si.control and si.CHM1_1) cells demonstrated CHM1 to clearly inhibit endothelial differentiation potential (scale bar 0.5 mm). (B) Analysis of osteolysis of A673 sh.CHM1 and negative controls (sh.control) in an orthotopic bone xenotransplantation model (five to eight mice per group). Affected bones were assessed by histology (TRAP staining, scale bar 0.25 mm or 0.05 mm). Left panel: quantitative summary of the average number of osteoclasts (mm^2^) in unaffected bone marrow, tumor samples, and attached to the bone in tumor tissues (bone). Data are mean ± SEM of at least two independent samples (at least 40 segments counted); *t*‐test. Right panel: Representative pictures are shown. CHM1 knockdown significantly enhanced the amount of TRAP‐positive osteoclasts attached to the bone (b) in the area of tumor (arrow) and thus increased the osteolytic phenotype. (C) Different ES cell lines with constitutive CHM1 knockdown and respective controls were analyzed by qRT‐PCR for expression of osteolytic genes such as *HIF1A*,*IL6*,*JAG1*, and *VEGF*. Data are mean ± SEM of two independent experiments; *t‐*test. *P < 0.05; **P < 0.005; ***P < 0.0005 (see 2.15. Statistical analyses).

Due to the well‐known antiangiogenic function of CHM1 (Hiraki *et al*., 1997; Yoshioka *et al*., 2006), we first tested the endothelial differentiation capacity of A673 and MHH‐ES1 cells either stable‐transfected with sh.CHM1 or sh.control or transiently with CHM1 or control siRNA, respectively (Fig. S1A), in a Matrigel matrix assay. As shown in Fig. 2A, CHM1 expression clearly inhibited the potential to form cellular tubes in ES cell lines irrespective of whether we investigated constitutive or transient knockdown of CHM1. Furthermore, CHM1 seems to be a key factor in chondrocyte development and proliferation inhibiting terminal chondrocyte differentiation to a hypertrophic phenotype during the process of endochondral ossification (Klinger *et al*., 2011; Shukunami and Hiraki, 2001). Thus, we incubated three ES cell lines stably transfected with sh.CHM1 and sh.control with specific differentiation media to induce chondrogenic or osteogenic differentiation. The differentiation potential was determined by qRT‐PCR using specific marker genes (Vater *et al*., 2011). As shown in Fig. S1B,C, the chondrogenic, and to a lesser extent the osteogenic, differentiation ability was significantly impaired after CHM1 knockdown. Based on these findings, we asked whether CHM1 might be important for the maintenance of an immature, chondrocytic phenotype of this tumor. Therefore, we analyzed the expression of different stem cell genes, namely *ATP‐binding cassette*,* subfamily G (WHITE)*,* member 2* (*ABCG2*; Szepesi *et al*., 2015; Zhou *et al*., 2001), *nanog homeobox* (*NANOG*; Mitsui *et al*., 2003), and *prominin 1* (*PROM1*; Katoh and Katoh, 2007), in ES cell lines with CHM1 knockdown and respective controls. As shown in Fig. S1D, suppression of CHM1 decreased the expression of *ABCG2* and *PROM1* compared to sh.control‐transfected cells, of which only PROM1, important for maintaining stemness and pluripotency, was down‐regulated down to 13.7% (32.9%), especially in A673 cells, after CHM1 knockdown at the protein level (Fig. S1E).

### 3.3 CHM1 represses osteomimicry of ES

Due to the particular effect of CHM1 especially on the chondrogenic differentiation potential of ES cells, we asked whether CHM1 may influence bone‐associated tumor growth of ES *in vivo*, as well. We injected constitutive sh.CHM1‐ or sh.control‐infected A673 cells (see Materials and methods 2.13) into the tibiae of immunodeficient Rag2^−/−^γc^−/−^ mice and analyzed bone infiltration and destruction by X‐ray radiography and histology. Many mice developed severe osteolytic lesions (both around 80%), regardless of whether mice were injected with A673 sh.control or sh.CHM1 cells (data not shown). However, the number of TRAP^+^ osteoclasts was significantly increased within bone tissue, but decreased within the tumor tissue in sh.CHM1 samples as compared to negative controls (Fig. 2B). A similar experiment with TC‐71 sh.CHM1 and sh.control cells could confirm these findings, even though only few mice (40%) developed a tumor regardless of whether injecting TC‐71 sh.CHM1 or sh.control cells (Fig. S2).

The increased osteolytic phenotype in bone tissue after CHM1 knockdown might result in better localization to bone in combination with a change in the expression pattern of cancer cells, also known as osteomimicry. We determined the mRNA levels of different genes known to be associated with osteolysis. As shown in Fig. 2C, CHM1 knockdown significantly increased the expression levels of osteolytic genes such as *hypoxia‐inducible factor 1*,* alpha subunit* (*HIF1A*), *interleukin 6* (*IL6*), *jagged 1* (*JAG1*), and *vascular endothelial growth factor receptor 1* (*VEGF*) (Weilbaecher *et al*., 2011), which may further increase the osteolytic and malignant activity within bone observed here (Fig. 2B).

### 3.4 CHM1 enhances proliferation in ES

**Figure 3:**
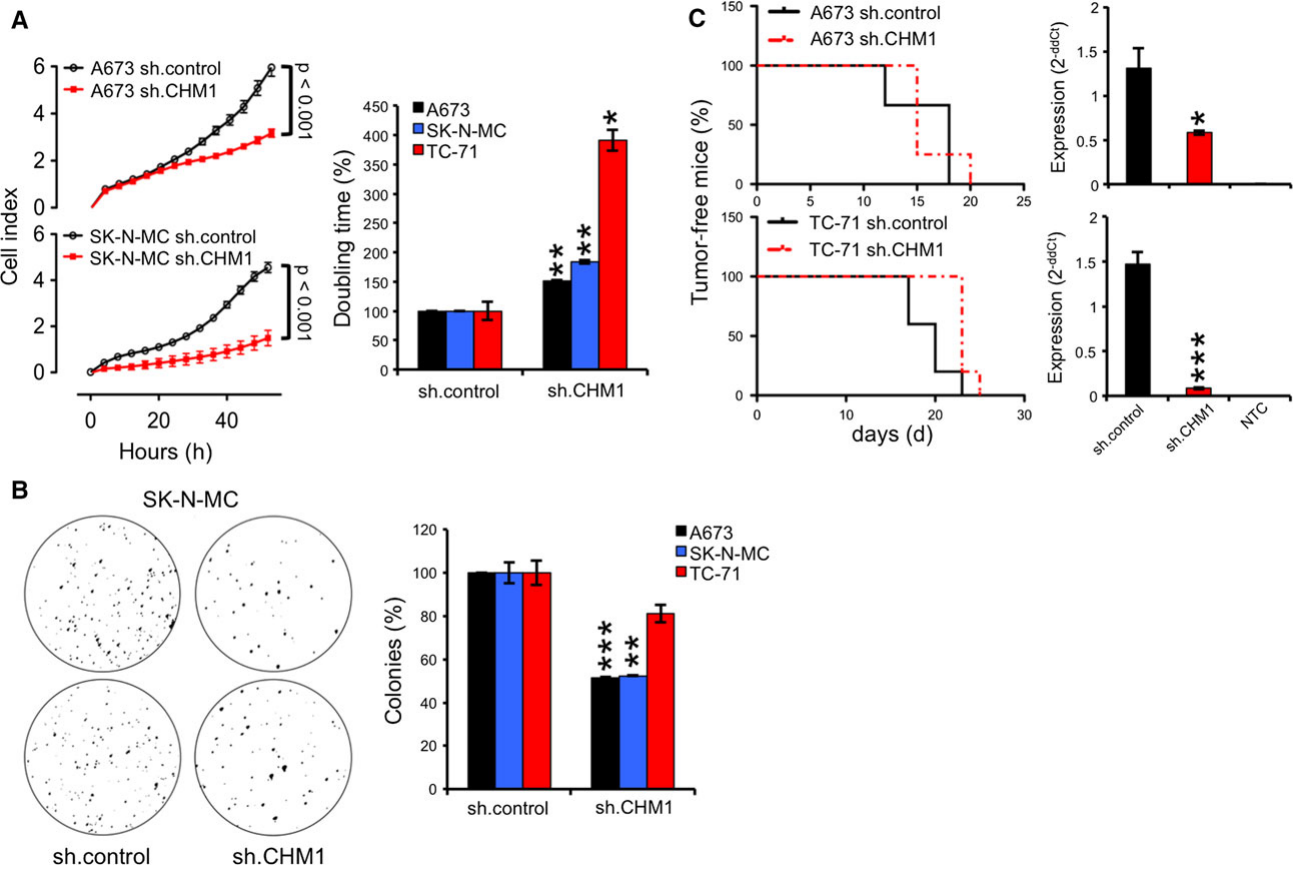
CHM1 delayed proliferation in ES *in vitro*. (A) Analysis of contact‐dependent growth of constitutively sh.CHM1‐ and sh.control‐infected ES cell lines with xCELLigence. Left panel: Cellular impedance was measured every four hours (relative cell index). Data are mean ± SEM (hexaplicate/group); *t*‐test. Right panel: doubling time of constitutive A673, SK‐N‐MC, and TC‐71 CHM1 shRNA infectants. Data are mean ± SEM of two independent experiments/cell line (hexaplicate/group); *t*‐test. B. Effect of CHM1 knockdown on anchorage‐independent growth in A673, SK‐N‐MC, and TC‐71 cells using methylcellulose matrices. Left panel: A representative experiment with SK‐N‐MC cells was shown as macrograph. Right panel: The average number of colonies of at least two different experiments with three different ES cell lines was shown after stable CHM1 suppression. (C) Left panel: evaluation of tumorigenicity of constitutive A673 and TC‐71 CHM1 shRNA infectants in immunodeficient Rag2^−/−^γc^−/−^ mice (3–5 mice per group). Right panel: post *ex vivo* CHM1 expression using qRT‐PCR. Data are mean ± SEM,* t*‐test. *P < 0.05; **P < 0.005; ***P < 0.0005 (see 2.15. Statistical analyses).

To further analyze the impact of CHM1 overexpression on the pathogenesis and malignancy of ES, we next examined the effect of CHM1 on *in vitro* proliferation using an xCELLigence‐based proliferation assay. As shown in Fig. 3A, constitutive down‐regulation of CHM1 significantly decreased contact‐dependent growth of all three ES cell lines investigated without affecting the cell cycle (Fig. S3). Interestingly, CHM1 similarly enhanced colony formation on methylcellulose matrices in A673, SK‐N‐MC, and TC‐71 cells *in vitro* (Fig. 3B). Subsequently, we analyzed whether CHM1 affects *in vivo* tumorigenicity of ES, too. We injected stably transfected A673 and TC‐71 cells with sh.CHM1 and sh.control subcutaneously into the inguinal region of immunodeficient Rag2^−/−^γc^−/−^ mice and analyzed local tumor growth. However, in contrast to *in vitro* proliferation, suppression of CHM1 only marginally delayed local tumor growth *in vivo* (Fig. 3C).

### 3.5 CHM1 enhances invasiveness and metastasis in ES

**Figure 4:**
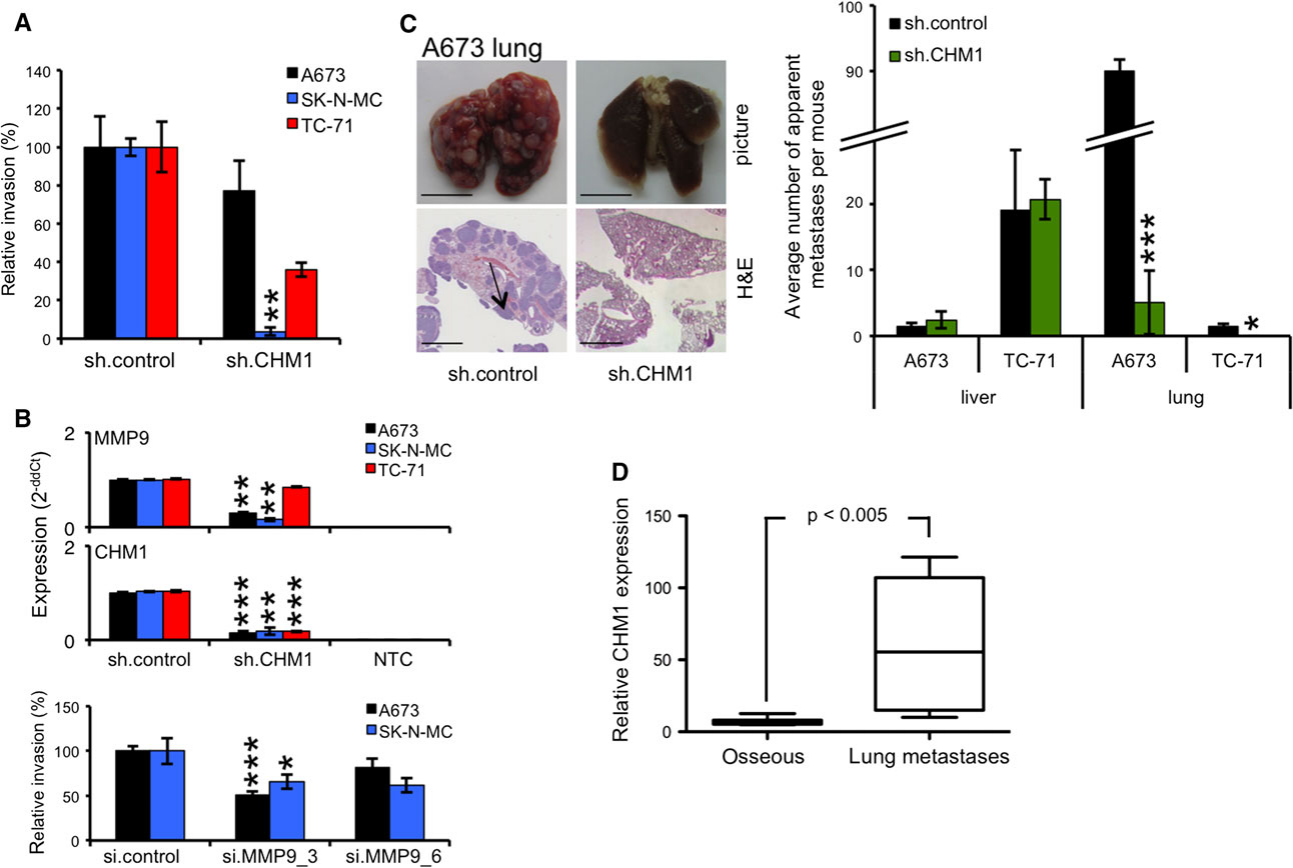
CHM1 enhances metastasis in ES *in vivo*. (A) Analysis of invasiveness of ES cell lines through Matrigel after transfection with specific CHM1 shRNA constructs. Data are mean ± SEM of two independent experiments; *t*‐test. (B) Upper panel: qRT‐PCR of *MMP9* expression after stable CHM1 knockdown. Data are mean ± SEM of two independent experiments; *t*‐test. Lower panel: analysis of the invasive potential of A673 and SK‐N‐MC cells after transient transfection with two specific *MMP9* siRNAs 48 h before seeding. Data are mean ± SEM;* t*‐test. (C) Analysis of metastasis using A673 and TC‐71 cells with stable CHM1 suppression and respective controls (four to five mice per group). Left panel: Representative lungs with corresponding H&E staining of A673‐injected mice are shown (scale bar 5 or 2 mm). Right panel: Average number of apparent metastases per mouse in lung and liver tissues is illustrated; *t*‐test. (D) DotBlot of relative CHM1 expression in ES osseous tumor samples compared to ES lung metastases samples using microarray analysis of 14 patient tumor samples. *P < 0.05; **P < 0.005; ***P < 0.0005 (see 2.15. Statistical analyses).

Invasiveness and metastasis are important hallmarks of cancer (Hanahan and Weinberg, 2011). Therefore, we tested three ES cell lines with constitutive CHM1 knockdown and respective controls in a Matrigel invasion assay. Stably silenced CHM1 ES cell lines showed a clear reduction in invasion down to 4% in SK‐N‐MC cells compared to control cells (Fig. 4A). As previously reported by our group, matrix metallopeptidases (MMPs) appear to be important for ES invasiveness (Grunewald *et al*., 2012; Hauer *et al*., 2013; Richter *et al*., 2013). Thus, we next examined the mRNA expression of *MMP1*,* MMP7*, and *MMP9* after CHM1 knockdown. As shown in Fig. 4B, suppression of CHM1 clearly reduced mRNA levels of *MMP9*, in contrast to *MMP1* and *MMP7* (Fig. S4A). Simultaneously transient MMP9 knockdown significantly decreased the amount of cells crossing the Matrigel, albeit not as strong as observed after CHM1 suppression (Fig. 4A,B bottom), indicating additional factors involved.

Finally, we investigated the metastatic potential of ES cells constitutively transfected with sh.CHM1 and sh.control in immunodeficient Rag2^−/−^γc^−/−^ mice. Even though there is no difference in cell size and there is only a minimal increase in granularity between sh.CHM1 and sh.control cells (Fig. S4B), suppression of CHM1 significantly reduced the number of lung metastases after inoculation with A673 cells (Fig. 4C). However, no clear differences were observed for liver metastases for these cells (Fig. 4C, right). These results were confirmed with TC‐71 cells; while no lung metastases were observed after CHM1 suppression, the number of liver metastases was not affected (Fig. 4C). Interestingly, modulation of angiogenesis did not seem to contribute to ES metastasis in our *in vivo* mouse model. Although CHM1 clearly inhibited the endothelial differentiation potential *in vitro* (Fig. 2A) and *in vivo*, no differences in angiogenesis were observed as demonstrated by CD31 and Mac‐3 staining of different lung and liver tumor samples (Fig. S4C,D).

To further determine the relevance of these results in the clinical setting, we analyzed samples from 14 patients with ES. Interestingly, microarray analysis revealed a significantly higher expression of CHM1 (*P*‐value < 0.005) in tumor samples derived from lung metastases than from different local relapses in bone localizations (Fig. 4D).

## 4 Discussion

The current study investigated the role of CHM1 for the biology and pathology of ES. We observed that EWS‐FLI1 specifically induced CHM1 expression.

CHM1 is a known antiangiogenic factor, which has been demonstrated to play a role in bone development and to be expressed in growth plate cartilage of hypertrophic and calcified zones (Hiraki *et al*., 1997; Miura *et al*., 2014; Yoshioka *et al*., 2006). Previously, we have shown that reduced tumor perfusion is associated with resistance and poor prognosis in ES (Dunst *et al*., 2001). CHM1 influences endochondral ossification as well as chondrocyte development and proliferation (Klinger *et al*., 2011; Shukunami and Hiraki, 2001). Its function may be mediated by its secreted form or by its intracellular effect on different pathways, respectively (Mera *et al*., 2009). ES cells secrete CHM1 as demonstrated via ELISA, but we have no direct information on potential membrane‐bound forms as available antibodies so far do not work reproducibly in western blot analysis (data not shown). However, following RNA interference, we observed CHM1 to affect endothelial, as well as chondrocytic, differentiation potential of ES, presumably via its intracellular activity. Because CHM1 maintains a more undifferentiated chondrocytic phenotype and represses endothelial differentiation of ES, we further investigated the expression of several stem cell genes. Although we did not observe a distinct phenotype, we could show that CHM1 enhanced the expression of *ABCG2* and *PROM1*. ABCG2 is expressed in a wide variety of stem cells (Zhou *et al*., 2001), while PROM1 is so in embryonic and adult as well as cancer stem cells and maintains stem cell properties by suppressing differentiation (Katoh and Katoh, 2007). ABCG2, in addition, seems to be a good marker for stem cells with enhanced osteogenic and chondrogenic differentiation potential (Szepesi *et al*., 2015). Remarkably, Tanaka *et al*. (2014) recently demonstrated that cells present in the embryonic superficial zone of long bones and of osteo‐chondrogenic origin are possible ES progenitor cells. Furthermore, epigenetic suppression of CHM1 in malignant tumor of bone such as osteosarcoma (Aoyama *et al*., 2004) is supportive for its presumed role maintaining an immature chondrocytic phenotype in ES.

While investigating how CHM1 influences tumor growth in our orthotopic xenograft mouse model (Hauer *et al*., 2013), we observed that overall tumor growth was relatively unaffected although an increase in TRAP^+^ osteoclasts in bone tissue following CHM1 suppression was detected. In line with this observation, we noticed an increased expression of malignancy‐promoting/osteolytic genes after CHM1 knockdown in ES cells, which might enhance aggressiveness and result in better localization to bone in combination with a change in the expression pattern of cancer cells, also known as osteomimicry. Expression of the transcription factor HIF1A by tumor cells inhibits osteoblast differentiation and enhances the differentiation and maturation of osteoclasts, in part via VEGF induction (Dunn *et al*., 2009; Hiraga *et al*., 2007; Weilbaecher *et al*., 2011). Furthermore, Guan *et al*. demonstrated that VEGF increases RANKL promoter activity in ES, leading to induced bone lysis (Guan *et al*., 2009), which may explain the increased osteolytic phenotype of ES after CHM1 knockdown in our osteotropic tumor model as observed here. In addition, JAG1, a potent downstream mediator of TGFB1, which promotes osteolysis in breast cancer cells by activating the NOTCH signaling pathway, leads to increased IL6 expression (Sethi *et al*., 2011; Tao *et al*., 2011). However, in ES, NOTCH signaling is switched off via EWS‐FLI1‐mediated repression (Ban *et al*., 2008; Bennani‐Baiti *et al*., 2011). IL6 is a pro‐proliferative cytokine, which promotes tumor growth (Ara *et al*., 2009) and is enhanced after CHM1 suppression in ES cells (Fig. 2C). Another prominent example with regard to osteomimicry observed here was the expression of *OPN*, which also increased after CHM1 knockdown especially in A673 cells (Fig. S1C). OPN is normally expressed by osteoclasts and facilitates attachment of osteoclasts to the bone matrix (Reinholt *et al*., 1990). Moreover, OPN is known to be secreted by tumor cells and promotes bone marrow cell recruitment and tumor formation in bones (Anborgh *et al*., 2010; Weilbaecher *et al*., 2011). Overall, these results may provide hints that CHM1 may balance a certain level of chondro‐osseous differentiation capability and supports stronger CHM1 expression in lung metastases compared to bone samples of patients with ES, as observed here.

Further analysis of ES malignancy revealed that CHM1 significantly enhances contact‐dependent as well as contact‐independent growth of different ES cell lines *in vitro*, but only marginally influences local tumor growth in xenograft mice after subcutaneous injection. Presumably, the CHM1‐mediated growth advantage *in vitro* may be reduced by a poorer supplement/support of tumor growth *in vivo* due to the known antiangiogenic function of this glycoprotein (Hiraki *et al*., 1997; Yoshioka *et al*., 2006).

Additionally, we clearly observed that CHM1 enhances *in vitro* invasiveness and significantly increased the mRNA expression of *MMP9* in different ES cell lines. In previous studies (Grunewald *et al*., 2012; Hauer *et al*., 2013; Richter *et al*., 2013), we demonstrated MMP1 to be the most important factor influencing ES invasiveness *in vitro* and *in vivo*. However, these results were not confirmed after knockdown of CHM1. Transient suppression of MMP9 clearly reduced the invasive potential of ES cells, as well, introducing MMP9 as another important factor in ES invasiveness. This observation is confirmed by different publications identifying MMP9 as a crucial factor associated with invasion in other tumor entities, such as breast and prostate cancer (Bin Hafeez *et al*., 2009; Wang *et al*., 2011). In line with these findings, *in vivo* knockdown of CHM1 mainly suppressed the development of lung metastases of different ES cells investigated in our mouse model, indicating CHM1 to be important for the development of lung but not for liver or bone metastases. These results were complemented by clinical data reinforcing a role of CHM1 for ES invasiveness and metastasis especially to lung tissues (Fig. 4D).

In summary, our results indicate that CHM1 preserves the immature chondrocytic phenotype of this disease and enhances clonality as well as invasiveness and the metastatic potential especially for lung metastasis *in vivo*, thereby promoting the malignant potential of this disease.

## Author contributions

KvH, SG, OS, DS, AF, and GHSR performed experiments. KvH, CMT, TH, and GHSR analyzed data. JCW, FN, and IE carried out pathology assessments and IHC analyses. SB and GHSR initiated the project. PS provided key insights into data interpretation. KvH and GHSR wrote the manuscript.

## Supporting information


**Fig. S1.** CHM1 maintains an undifferentiated phenotype of ES.

**Fig. S2.** *In vivo* bone invasion and osteolysis.

**Fig. S3.** Cell cycle distribution analyses.

**Fig. S4.** CHM1 knock down does not influence *in vivo* angiogenesis.

**Doc S1.** Materials and methods.
