# Supplementary figures and images for: The endochondral bone protein CHM1 sustains an undifferentiated, invasive phenotype, promoting lung metastasis in Ewing sarcoma
Source: Mol Oncol. 2017 Aug 21;11(9):1288–301. doi: 10.1002/1878-0261.12057 (PMC5579336; doi:10.1002/1878-0261.12057)

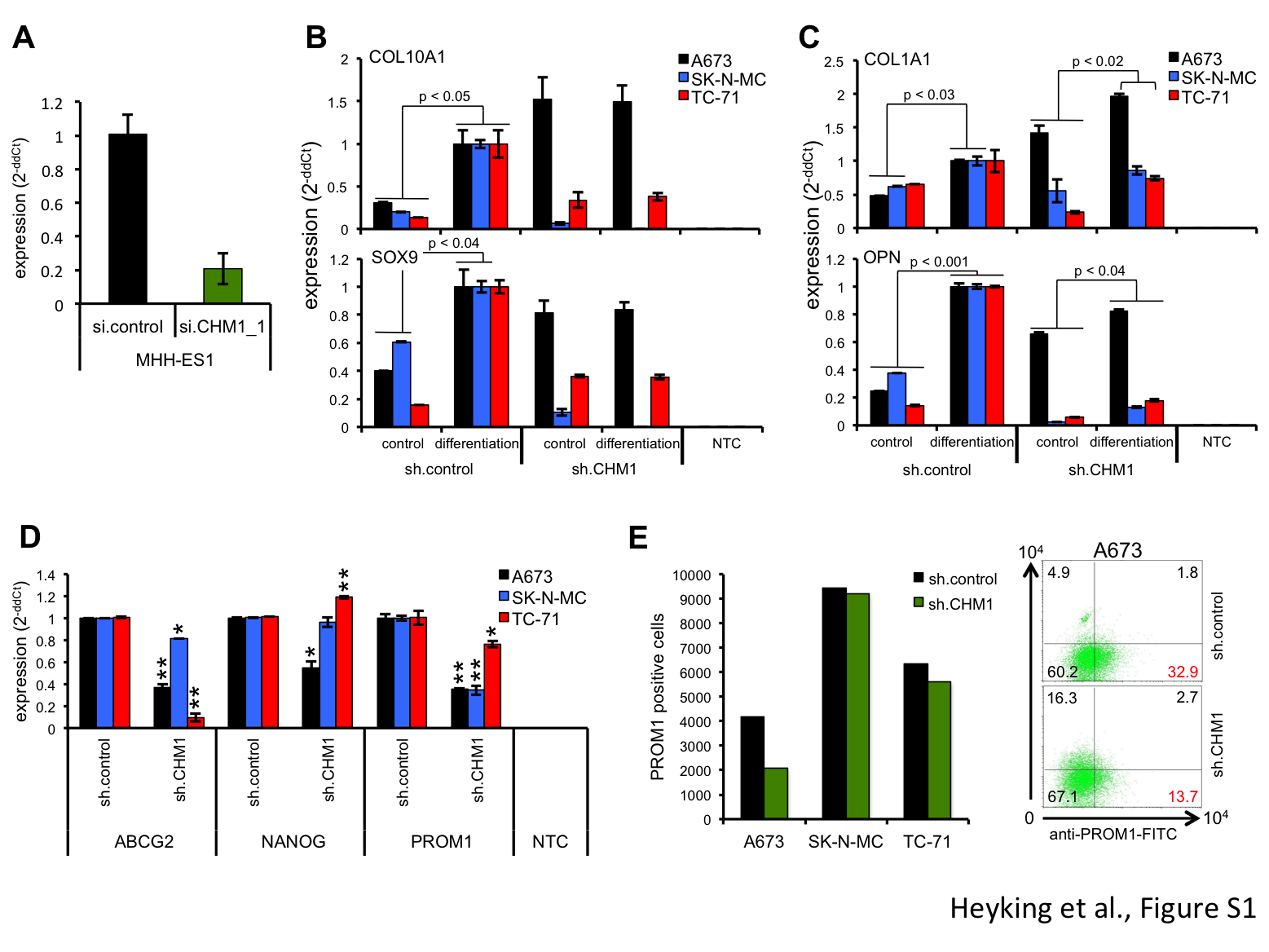

Supplement: Supplementary file 1 — Fig. S1. CHM1 maintains an undifferentiated phenotype of ES. [file MOL2-11-1288-s001.tif]

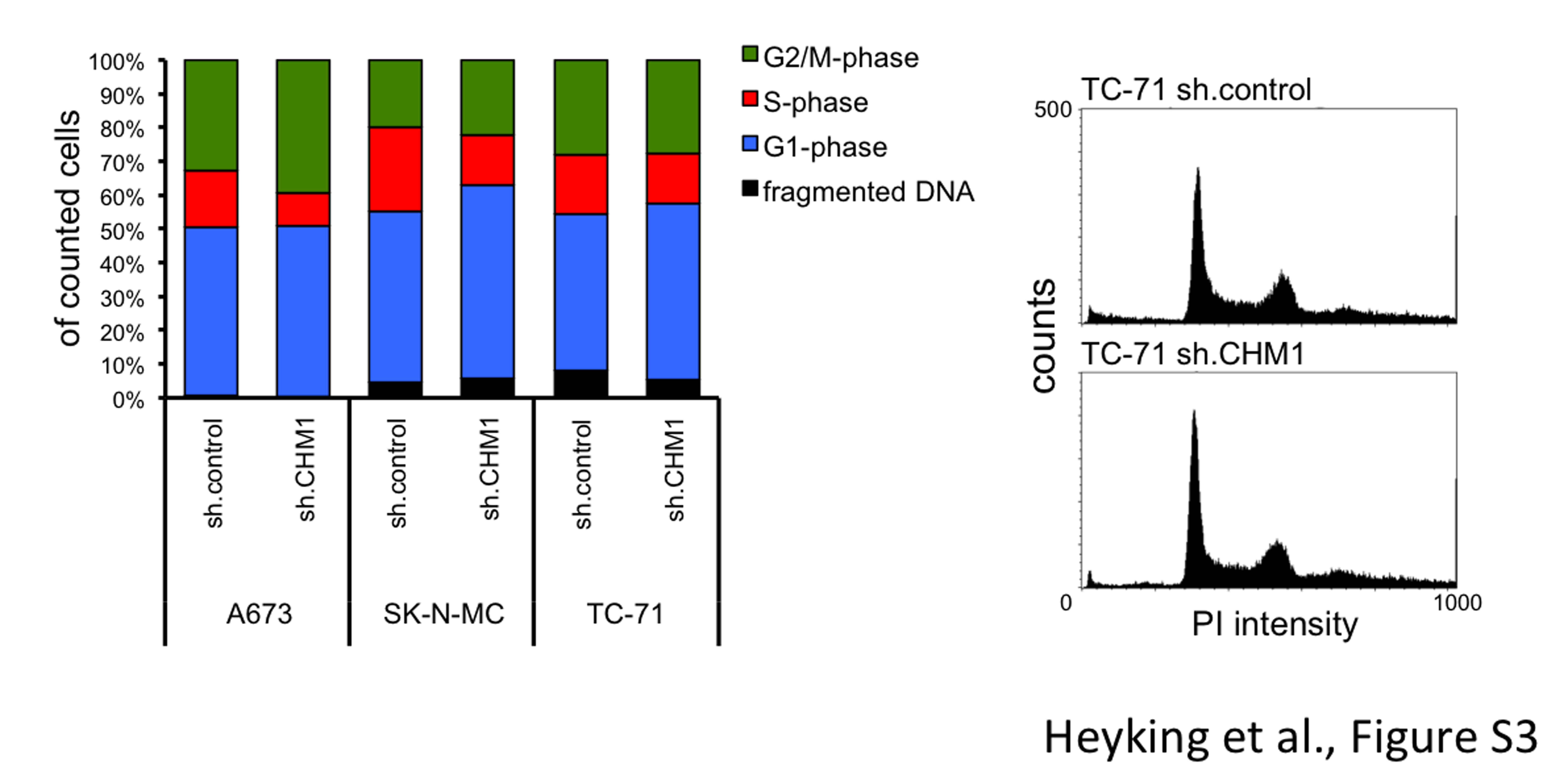

Supplement: Supplementary file 3 — Fig. S3. Cell cycle distribution analyses. [file MOL2-11-1288-s003.tif]

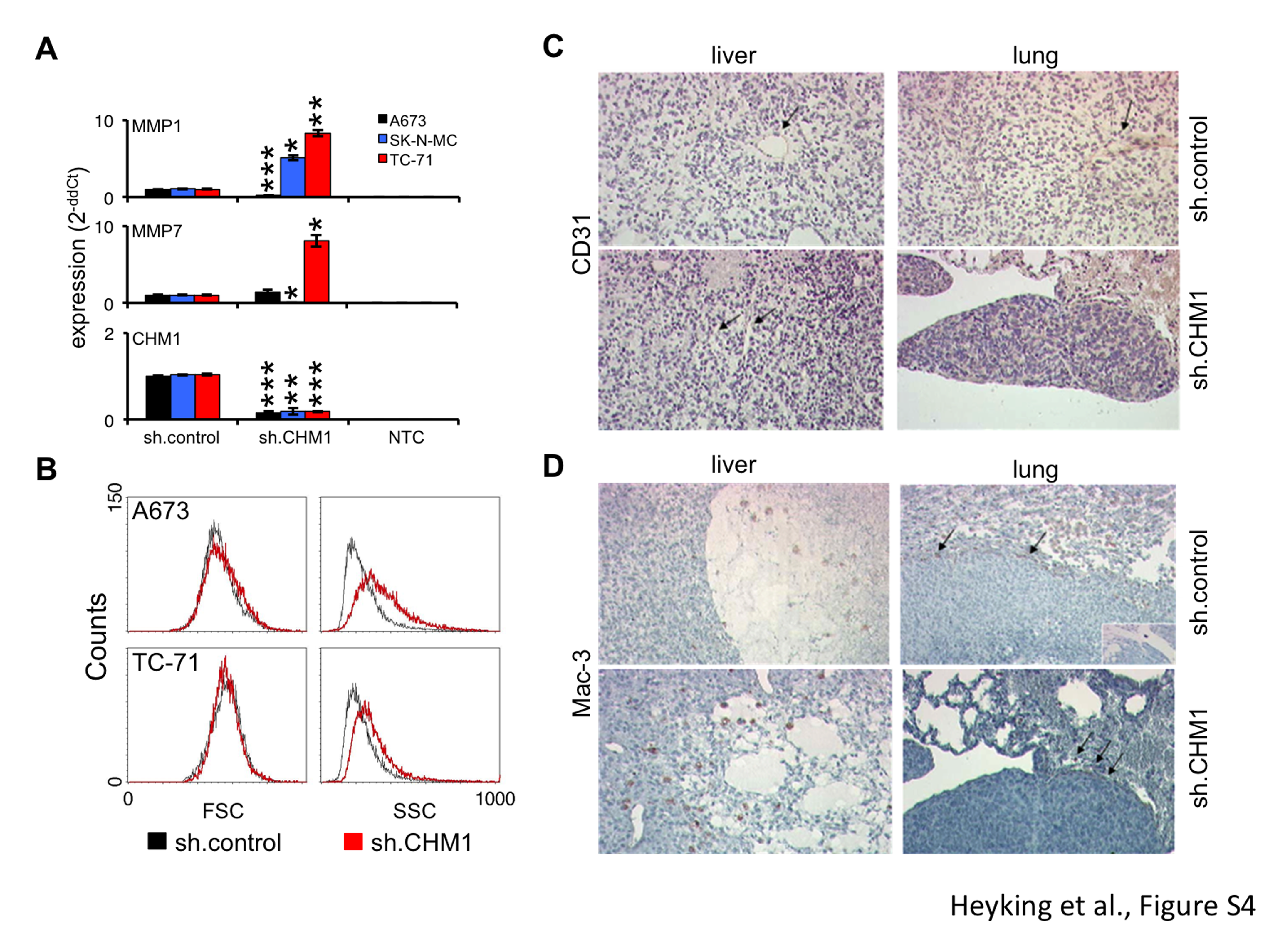

Supplement: Supplementary file 4 — Fig. S4. CHM1 knock down does not influence in vivo angiogenesis. [file MOL2-11-1288-s004.tif]
