# Supplementary material for: The endochondral bone protein CHM1 sustains an undifferentiated, invasive phenotype, promoting lung metastasis in Ewing sarcoma
Source: Mol Oncol. 2017 Aug 21;11(9):1288–301. doi: 10.1002/1878-0261.12057 (PMC5579336; doi:10.1002/1878-0261.12057)
Supplement: Supplementary file 5 — Doc S1. Materials and methods. [file MOL2-11-1288-s005.docx]

**APPENDIX A: Supplementary data and figures**

**A.1. SUPPLEMENTARY MATERIALS AND METHODS**

**A.1.1. Small interfering RNAs used.** siRNAs for EWS-FLI1 were synthesized at MWG Biotech and correspond to published sequences (Dohjima et al., 2003). All other siRNAs were purchased from Qiagen. siRNA CHM1_1 5’-CGAAGGAAUCUGUUGUAUATT-3’ (sense) and 5’-UAUACAACAGAUUCCUUCGTG-3’ (antisense); siRNA EWS-FLI1_1 5’-GCUACGGGCAGCAGAACCCUU-3’ (sense) and 5’-AAGGGUUCUGCUGCCCGUAGC-3’ (antisense); siRNA EWS-FLI1_2 5’-GCAGAACCCUUCUUAUGACUU-3’ (sense) and 5’-GUCAUAAGAAGGGUUCUGCUUU-3’ (antisense); siRNA MMP9_3 5’-GACGCACGACGUCUUCCAGUA-3’ (sense) and 5’-UACUGGAAGACGUCGUGCGUC-3’ (antisense); siRNA MMP9_6 5’-ACGGCUUGCCCAGGUGCAGUA-3’ (sense) and 5’-UACUGCACCUGGGCAAGCCGU-3’ (antisense) and control non silencing siRNA (si.control) 5’-UUCUCCGAACGUGUCACGU-3’ (sense) and 5’-ACGUGACACGUUCGGAGAA-3’ (antisense).

**A.1.2. Short hairpin RNA coding oligonucleotides.** The following oligonucleotides were used: CHM1-forward 5’-GATCCGCGAAGGAATCTGTTGTATATTCAAGAGATATACAACAGATTCCTTCGCTTTTTTCTAGAG-3’ and CHM1-reverse 5’-AATTCTCTAGAAAAAAGCGAAGGAATCTGTTGTATATCTCTTGAATATACAACAGATTCCTTCGCG-3’; and control-forward 5’-GATCCGTTCTCCGAACGTGTCACGTTTCAAGAGAACGTGACACGTTCGGAGAACTTTTTTCTAGAG-3’ and control-reverse 5’-AATTCTCTAGAAAAAAGTTCTCCGAACGTGTCACGTTCTCTTGAAACGTGACACGTTCGGAGAACG-3’ (all Metabion International AG).

**A.1.3. Primers and assays used for qRT-PCR.** For EWS-FLI1 detection, the following primers 5’-TAGT-TACCCACCCCAAACTGGAT-3’ (sense), 5’-GGGCCGTTGCTCTGTATTCTTAC-3’ (antisense) and probe 5’-FAM CAGCTACGGGCAGCAGAACCCTTCTT-TAMRA-3’ were designed. Inventoried TaqMan Gene Expression Assays (Life Technologies) were used for the genes *ABCG2* (Hs00184979_m1), *CHM1* (Hs00993254_m1), *COL1A1* (Hs00164004_m1), *COL10A1* (Hs00166657_m1), *GAPDH* (Hs99999905_m1), *HIF1A* (Hs00153153_m1), *IFITM1* (Hs00705137_s1), *IL6* (Hs00985639_m1), *ISG15* (Hs00192713_m1), *JAG1* (Hs01070032_m1), *MMP1* (Hs00899658_m1), *MMP7* (Hs01042796_m1), *MMP9* (Hs00234579_m1), *NANOG* (Hs02387400_g1), *OPN* (Hs00959010_m1), *PROM1* (Hs01009250_m1), *SOX9* (Hs00165814_m1), *VEGF* (Hs00176573_m1).

**A.1.4. Primers used for ChIP**

CHM1_-1894_fwd AGAACCACTTGAACCTGGGAG

CHM1_-1894_rev GTCTTGCTCTATCGCCCAGG

CHM1_-1060_fwd CCTATCACTCTCCCAATCCTCC

CHM1_-1060_rev AGGGGAGAAGTGGAGGAGAC

CHM1_-1036_fwd TCCCACAGCCCAGGAATTG

CHM1_-1036_rev CATGATGACCTGGGGCTGG

CHM1_-992_fwd ACTTTCTCCTTCTGCAGTCCTG

CHM1_-992_rev GGTGAGGTGTAAGTGAGGAGC

CHM1_-665_fwd TGGAACTGGAAGATGGCATTTC

CHM1_-665_rev CAATGGCAAAGCACACACAC

CHM1_-240_fwd CCACCCCAACACACACCTAC

CHM1_-240_rev CAATTAGCCATGCACCGGAG

EZH2_-1081_fwd GACACGTGCTTAGAACTACGAACAG

EZH2_-1081_rev TTTGGCTGGCCGAGCTT

**A.1.5. Differentiation assays**

For testing of chondrogenic and osteogenic cell differentiation, cells were cultured in specific differentiation media (STEMPRO Chondrogenesis or Osteogenesis Differentiation Kit, GIBCO, Life Technologies) for two or three weeks at 37 °C / 5 % CO_2_ according to the manufacturer’s instructions. To validate differentiation efficacy, expression of the well-known chondrogenic marker genes, *collagen, type X, alpha-1 (COL10A1)* and *SRY-box 9* (*SOX9)* or osteogenic marker genes, *collagen, type I, alpha-1 (COL1A1)* and *secreted phosphoprotein 1 (SPP, OPN)* was monitored by qRT-PCR (Vater et al., 2011).

**A.1.6. Flow cytometry**

To detect protein levels, cells were washed twice in 1x Dulbecco’s phosphate buffered saline (PBS) and re-suspended in 40 μl staining buffer containing 10 μl human IgG (100 μg/ml). After an incubation time of 10 min on ice, 2 μl of the anti-PROM1 antibody (CD133/2 (293C3) (MACS Miltenyi Biotec)) were added and cells were incubated for further 30 min on ice. Cells were then washed with 100 μl staining buffer, re-suspended in 200 μl 1x PBS and fluorescence of cells was immediately analyzed using a FACSCalibur flow cytometer (BD Biosciences) and CellQuest software (BD Biosciences). At least 10,000 gated events per sample were recorded.

For cell cycle analysis, cells were fixed in ice-cold 70 % ethanol at -20 °C overnight and stained with propidium iodide (50 mg/ml) plus RNase (5 mg/ml) in 1 x PBS. The fluorescence of cells was measured using a FACSCalibur flow cytometer (BD Biosciences) and analyzed by Cellquest software (BD Biosciences). At least 10,000 gated events per sample were recorded.

Cell size and granularity of different ES cell lines upon stable CHM1 knock down compared to controls were analyzed by flow cytometry measuring forward (FSC) and side scatter (SSC) with a FACSCalibur flow cytometer (BD Biosciences) and Cellquest software (BD Biosciences). At least 10,000 gated events per sample were recorded.

**A.1.7. IHC staining**

Murine organs were fixed in phosphate buffered 4 % formaldehyde and embedded in paraffin. Three to five µm thick sections were stained with antibodies against the angiogenesis marker CD31 and Mac-3. All sections were reviewed and interpreted by I.E.

**A.2. SUPPLEMENTARY REFERENCES**

Dohjima, T., Lee, N.S., Li, H., Ohno, T., Rossi, J.J., 2003. Small interfering RNAs expressed from a Pol III promoter suppress the EWS/Fli-1 transcript in an Ewing sarcoma cell line. Mol Ther 7, 811-816.

Vater, C., Kasten, P., Stiehler, M., 2011. Culture media for the differentiation of mesenchymal stromal cells. Acta biomaterialia 7, 463-477.

**A.3. SUPPLEMENTARY FIGURE LEGENDS**

**A.3.1. Figure S1 - CHM1 maintains an undifferentiated phenotype of ES. A.** RNA interference of CHM1 expression. si.CHM1_1 represents the specific siRNA (si.control: non silencing siRNA). Results of qRT-PCR 48 hours after transfection are shown. Data are mean ± SEM of two independent experiments; t-test. **B.** Chondrogenic differentiation of ES cell lines constitutively infected with specific CHM1 and nonsense shRNA constructs was analyzed by the expression of specific chondrogenic marker genes *COL10A1* and *SOX9* using qRT-PCR. Data are mean ± SEM of two independent experiments; t-test (p: p-value). **C.** Analysis of osteogenic differentiation of ES shRNA infectants. Expression of the early (*COL1A1*) and late (*OPN*) marker genes was analyzed by qRT-PCR. Data are mean ± SEM of two independent experiments; t-test (p: p-value). **D.** mRNA levels of *ABCG2*, *NANOG* and *PROM1* in ES cell lines constitutively transfected with sh.CHM1 and sh.control were analyzed by qRT-PCR. Data are mean ± SEM of three independent experiments; t-test. **E.** Flow cytometric analysis of ES cell lines stably transfected with CHM1 shRNA and respective controls. Cells were stained with an antibody against the stem cell marker prominin 1 (PROM1) and the total number of PROM1 positive cells in comparison to isotype controls is shown for A673, SK-N-MC and TC-71 cells and their derivatives. A representative dot plot is shown for A673 sh.CHM1 and sh.control cells.

**A.3.2. Figure S2 – *In vivo* bone invasion and osteolysis.** Analysis of osteolysis of TC-71 sh.CHM1 and negative controls (sh.control) in an orthotopic bone xenotransplantation model (5 mice/group, in each case 40 % developed a tumor). Affected bones were assessed by histology. **Left panel:** Quantitative summary of the average number of osteoclasts (mm^2^) in unaffected bone marrow, tumor samples and attached to the bone in tumor tissues (bone). Data are mean ± SEM (at least 20 segments counted); t-test. **Right panel:** Representative pictures are shown. CHM1 knock down significantly enhanced the amount of TRAP-positive osteoclasts attached to the bone (b) in the area of tumor (t) and thus increased the osteolytic phenotype (TRAP staining, scale bar 0,1 mm).

**A.3.3. Figure S3 - Cell cycle distribution analyses.** Cell cycle distribution analyses of CHM1 shRNA infectants in different ES cell lines by propidium iodine staining and flow cytometry. A representative histogram is shown for TC-71 sh.CHM1 and sh.control cells.

**A.3.4. Figure S4 - CHM1 knock down does not influence *in vivo* angiogenesis.** **A.** Expression levels of *MMP1* and *MMP7* were analyzed in A673, SK-N-MC and TC-71 cells constitutively transfected with sh.CHM1 and sh.control using qRT-PCR. Data are mean ± SEM of two independent experiments; t-test. **B.** Cell size and granularity of A673 and TC-71 ES cell lines transfected with sh.CHM1 or sh.control were analyzed using flow cytometry; but no significant differences between CHM1 knock down cells and controls were detected. **C.** Immunohistochemistry of paraffin embedded lung and liver sections from Rag2^-/-^γc ^-/-^ mice after intravenous injection of A673 shRNA infectants. CD31 staining of liver and lung sections is shown (magnification 20 x). Arrows indicate thin-walled vessels. **D.** Mac-3 staining of paraffin embedded lung and liver sections of Rag2^-/-^γc^-/-^ immune deficient mice after intravenous injection of A673 shRNA infectants (magnification 10 x). Arrows indicate tumour-infiltrating macrophages (brown). Blue background staining due to treatment of H&E stained sections prior to CD31 staining.
